# The development of the Pan American Health Organization digital health specialist on alcohol use

**DOI:** 10.3389/fdgth.2022.948187

**Published:** 2022-10-26

**Authors:** Maristela G. Monteiro, Daniela Pantani, Ilana Pinsky, Thiago Augusto Hernandes Rocha

**Affiliations:** ^1^ Senior Advisor on Alcohol, Department of Noncommunicable Diseases and Mental Health, Pan American Health Organization, Washington, DC, United States; ^2^ International Consultant, Department of Noncommunicable Diseases and Mental Health, Pan American Health Organization, Washington, DC, United States; ^3^ Advisor on Public Health Data Analysis, Department of Health Analysis, Metrics and Evidence, Pan American Health Organization, Washington, DC, United States

**Keywords:** alcohol use, alcohol risk assessment, digital health worker, artificial intelligence, health literacy, digital health

## Abstract

**Introduction:**

On 19 November 2021 the Pan American Health Organization (PAHO) developed and deployed the first-ever digital health worker dedicated to alcohol-related topics, named Pahola. This paper describes this developmental process and the first results of its uptake and interactions with the public.

**Methods:**

PAHO secured a non-exclusive worldwide license with a technology company to use their Human OS ecosystem, which enables human-like interactions between digital people and users *via* an application. Google Digital flow ES was used to develop the conversations of Pahola on topics related to alcohol and health, screening of alcohol risk using the AUDIT and providing a quit/cut back plan to users, along with additional treatment services and resources in each country of the Americas. A communication campaign was also implemented from launching date until 31 December 2021.

**Results:**

Pahola attracted good attention from the media, and potentially reached 1.6 million people, leading to 236,000 sessions on its landing page, mostly through mobile devices. The average time people effectively spent talking to Pahola was five minutes. Major dropouts were observed in different steps of the conversation flow.

**Discussion:**

Pahola was quickly able to connect to a large worldwide population with reliable alcohol information. It could potentially increase the delivery of SBI and improve alcohol health literacy. However, its preliminary results pointed to much needed changes to its corpus and on its accessibility, which are being currently implemented.

## Introduction

The COVID-19 outbreak had an enormous impact on the mental health of populations globally, including in the region of the Americas, and exposed the deficits in health care systems in addressing mental health problems (1). Before this pandemic, the treatment gap for alcohol use disorders was already the highest among all mental health conditions in Latin America (2). During the pandemic, alcohol consumption increased among those who were already drinking excessively (3). Alcohol sales quickly adapted to the closure of points of sale through the expansion of online sales and home deliveries (4). This situation potentially created a new norm for drinking at home, within the family and alone, with consequences that are yet to be assessed.

At the same time, COVID-19 accelerated the need for a rapid improve in the access to safe and effective health services (5). On the one hand, digital health solutions, such as telehealth and online trainings, became rapidly popular to ease the disruption of healthcare services and to continue delivering care at all levels of the system. On the other side, as people reached out to the internet and social media for resources and connection, a parallel increase in the amount of misinformation about alcohol and health outcomes occurred and, as in many others health topics, left the population without a trusted and reliable source of information on alcohol use, harms and support (6, 7).

The use of digital solutions to improve alcohol health literacy and scale up support for individuals to reduce their alcohol consumption, such as by expanding Screening and Brief Interventions (SBIs), has been discussed for many years (8–10). Among different emerging technologies, such as chatbots, apps, geotagging and many others, digital health agents have the potential to provide reliable and timely information quickly, assess and support behavioral change to reduce the harmful use of alcohol to millions of people around the world and in the Americas (8).

The delivery of SBI for individuals with risky drinking is among the key alcohol public health policies recommended to address noncommunicable diseases (11). A model for its implementation by non-specialists in primary health care was developed by the World Health Organization (WHO) in the 1980's. This model consisted of a screening instrument to access alcohol risk consumption (Alcohol Use Disorders Identification Test—AUDIT) linked to a brief intervention for those who exceed recommended drinking limits, and referral to treatment for people with a diagnosable alcohol condition (alcohol use disorder) or who do not respond to the brief intervention (12). Efficacy and effectiveness studies in primary care settings found a consistent decrease in the individual self-reports of drinking for a period after the intervention (13).

Nonetheless, large scale real-life implementation of SBI overall has shown to be complex and it presents several obstacles. Reviews suggest that even when substantial preparation effort is employed, including high-level training, many clinicians do not apply SBI (14–16). When they do employ it, they frequently do not do it as they were trained to, impacting the results negatively (17). The literature focused on implementation challenges points out that among the barriers frequently described are the reluctance of clinicians to initiate discussions about alcohol. This happens in part due to practical/time constraints, but also to alcohol-related stigma, and uncertainty about how to assist patients with more severe alcohol problems, including obstacles with referral to treatment (18–20).

Computer-based interventions have emerged as instruments to improve and scale up the implementation of SBI programs for risky drinkers (21). The several formats of digital SBI include access to websites for risky drinkers' self-care or with the assistance of a health professional, mobile-health solutions, and more recently, digital conversational agents based on artificial intelligence. The literature has shown that digital interventions seem to lower alcohol consumption in a similar level as face-to-face SBI interventions (21, 22).

Nevertheless, a changing paradigm, that seems to point to the potential increasing contribution of digital interventions, propose that SBI should broaden its scope (20). The efforts to integrate SBI into health care system could include macro-level population issues, with SBI being more directly linked to alcohol policy measures. As a result, it would not be restricted to discussing the “self-regulation of one's own drinking” but would invite the individual to think about other environmental stimuli, such as persuasion by ubiquitous marketing, low alcohol prices, easy accessibility, wide availability etc.

This integrative approach could include people's access to health information and to a broader understanding of social norms, attitudes and environmental factors affecting their own behaviors, fostering their motivation and capabilities to change their health outcomes effectively and positively, which is the general idea behind the concept of health literacy (23). When applied to alcohol, health literacy could comprehend different components, including how individuals obtain, process and understand different alcohol-related topics, including the knowledge on alcohol content and strength of various alcohol products, alcohol harms and the impact of marketing approaches on the decision to consume alcohol; how people process and deconstruct marketing and media messages that could influence their behaviors; how alcohol problems are accepted in societies; and how individuals can act collectively to demand the adoption and implementation of alcohol policies to control the harms caused by alcohol use (10).

In 2020, the WHO developed and deployed a digital conversational agent to provide information on COVID-19 and on tobacco cessation resources, named Florence (24), along with other chatbots *via* WhatsApp, Viber and Facebook. Based on these innovative initiatives and their potential positive impact if applied to alcohol, the Pan American Health Organization (PAHO) developed and deployed the first-ever conversational agent dedicated to alcohol-related topics, named Pahola, in 2021.

Conversational agents are computer programs designed to simulate human text or verbal behavior (25). They can interact with an end-user using natural language processing (NLP), providing customized feedback following a predefined topic. In terms of capabilities, the conversational agents can be categorized as simple or smart (25). Simple agents can only provide answers within predefined scripts, with the possibility of free interaction or inputs from the user side. On the other hand, smart conversational agents do not respond with preprepared answers but with adequate suggestions instead. This type of agent is developed using artificial intelligence solutions, which allows for broadening of the computer system's capacity through its learning from data (in this case conversations) without being explicitly programmed. Recent advances in the field of NLP and natural language understanding (NLU) have achieved unprecedent levels of accuracy. The use of the BERT (Bidirectional Encoder Representations from Transformers) presented state-of-the-art results in a wide variety of NLP tasks, including Question Answering (SQuAD v1.1), natural language inference (MNLI) (26). Despite the advances in the field of NLP, up to this moment, no initiative has been performed to integrate a conversational agent using BERT-based approaches to provide information regarding alcohol-related issues. Thus, PAHO was engaged in developing Pahola, aiming to shed light on the following question: Is it possible to integrate NLP cutting-edge technologies to develop a multilanguage conversational agent dedicated to addressing alcohol-related information capable of engaging end users in an anonymous context? This paper describes the developmental process and the preliminary results of its uptake and interactions with the public.

## Materials and methods

Within the implementation science framework, the present study is categorized as observational. It aims not to intervene with study participants but instead describes outcomes of interest and their antecedents in their natural context (27). The observation design was chosen considering its useful approach to evaluating the real-world applicability of evidence.

Seven technology components were needed to develop and deploy Pahola:

1. Facial sentiment analysis of end user video stream.
2. Autonomous animation of digital person with cognitive models.
3. 3D rendering cloud architecture
4. WebRTC video and audio streaming front-end SDK.
5. Natural language processing.
6. Speech to text conversion (STT).
7. Text to speech synthesis (TTS) module.

The facial sentiment analysis module is responsible for processing real-time gesturing. This feature enables Pahola to analyze what has been said *via* NLP and adds emotionally appropriate gesturing and behavior to their speech in real-time. The second module (autonomous animation) controls the technology behind the conversational agent rendering in real-time, including matching mouth movements to speech and facial expressions. This means the agent's expressions can be triggered by phrases and words to mimic personality tags and make them specific to the conversation.

Pahola exists in a 3D scene which is viewed through a virtual camera. Changing the way that the camera behaves to suit your content can result in a much more engaging experience. The third module is responsible for processing the 3D scene in which the conversational agent is inserted.

The WebRTC Video and Audio Streaming Front-end SDK (module 4) is responsible for organizing the information workflows between the user input by the end-user and the NLP processing engine. Audio utterances from the end user are transmitted *via* WebRTC for conversion to text. The resulting text is then directed to the NLP, to a phrase hints process. Every match between the text translated is associated with predefined phrases registered in Pahola's dialog three. Text responses generated by the NLP are first pre-processed for parsing of Emotional Markup Language (EML, means that the scripting language used within the NLP indicates the expression Pahola should manifest based on the intended experience), with the remaining static text processed by the TTS service (module 7). The final output is the voiced content uttered by Pahola.

The fifth module relies on the Google Dialogflow ES for NLP to understand the user's intention or inquiry by analyzing their utterance and select the proper pre-written response from a finite set of responses. This is the module performing the match between processed audio input provided by the end-user and the dialog three linked to the conversational agent. For integrating the NLP core processing engine with the predefined set of questions, we provided matching phrases as part of building the conversation (for example, variations of phrases provided by PAHO on alcohol related topics as well variations of responses to the same questions). From these phrases, the NLP generates more examples and learn over time from interacting with users.

Items 6, and 7 are NLP modules dedicated to processing audio signals produced by your user, as well as responding in a natural way. The STT service converts the audio said by the end user into machine readable text format. The TTS is responsible for Pahola's voice synthesis. Thus, it is the module responsible for converting predefined answers registered into Pahola's dialog three to the audio used to answer a question done by the end-user. The STT and TTS modules are provided by Google, through Dialogflow ES for NLP. It is relevant to highlight that we did not perform the NLP training of the model used to interpret the audio or text message provided by the user. These features were performed by the Google Dialogflow ES machine learning service. Our option to use the Google service was due to its unprecedent level of precision in terms of NLP and NLU tasks in a multilanguage context, using BERT. Further details of the training performance and level of precision achieved by this new encoder can be found in the work of Devlin et al., 2018 (26).

Each module described above performed a group of tasks to automatically process the audio or text inputs provided by the end-users. PAHO secured a non-exclusive worldwide license from a private company to use a software that enables interactions between a digital conversational agent and users *via* an application.

PAHO decided on digital conversational agent's gender, race, personality, tone of voice, apparent age, and clothing colors and style. The decisions were based on *ad hoc* informal consultations with about 30 staff from PAHO, as well as alcohol experts in several countries. The name Pahola was chosen to intentionally relate to PAHO's acronym, as to minimize the likelihood of a duplicate proper name from another digital person already patented. Pahola was developed in three languages for the first launching and deployment (English, Portuguese and Spanish).

PAHO also contracted a marketing company to jointly develop the use case ideation, persona, conversation design, and the NLP integration with basic EML that required the implementation of specific phrases that consistently trigger specific emotions by Pahola. PAHO provided technical support on the content development and the evidence-based information that were added in the corpus, as well as revised and approved all content.

After a welcome and rapport conversation with Pahola, which includes asking users if they consider their drinking below, above or average to increase awareness about how they perceive their alcohol use, the corpus included three branches the user could choose to start an interaction with Pahola: (1) alcohol and health topics (“alcohol effects branch”), including general information about the impact of alcohol on people's health and alcohol health literacy; (2) assessing user's own risk from drinking alcohol (“quiz branch”); and (3) seeking treatment resources to quit or reduce drinking (“quit branch”).

Around 100 different alcohol-related health themes were added to the alcohol effects branch. The themes covered: what a standard drink is; if drinking is healthy; if alcohol can cause cancer, heart disease, mental health problems; how alcohol affects physical performance; how it affects immunity; alcohol and COVID-19; alcohol and liver problems; alcohol and sleep; alcohol dependence; alcohol use and gender differences; alcohol and pregnancy; alcohol use among youth; alcohol and violence; alcohol and driving; and so on. They were selected non-systematically based on common misunderstandings about alcohol harms reported in social media channels and on the internet, as well as on *ad hoc* consultations with non-specialists, women in recovery from alcohol problems, alcohol researchers, PAHO staff and their family members, and young people in various countries.

For assessing the person's risk from drinking (quiz branch), the AUDIT questionnaire was incorporated into the conversation flow. If the results indicated a moderate or high risk for alcohol problems, Pahola would provide initial feedback and propose basic steps of a brief advice for quitting or reducing drinking (quit branch). In addition, a webpage was created with information on treatment services for each country in the Americas, as well as a link to Alcoholic Anonymous International, where users could find a mentor or an online group to join, anywhere in the world, in any of the PAHO official languages. The information compiled was limited to health services that did not have a cost to the user and/or were recommended by the government of each country.

**Figure 1:**
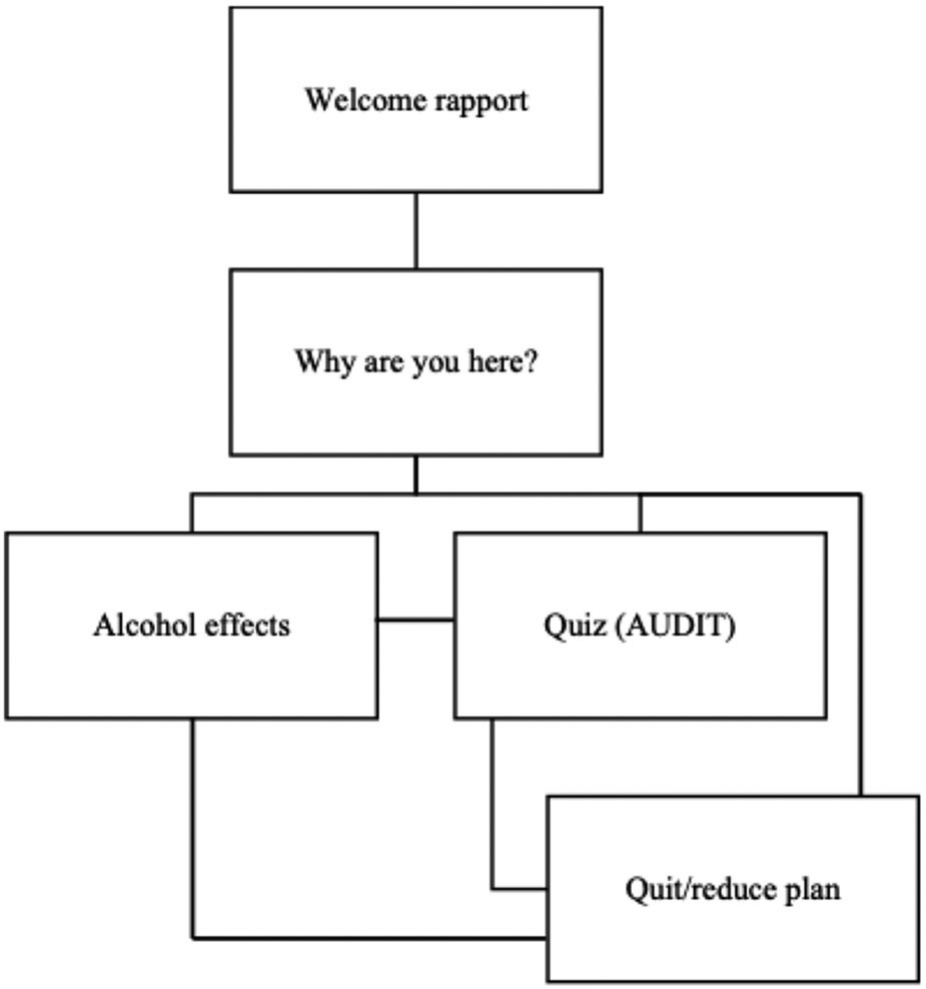
Summary of Pahola's dialog tree.

The conversation corpus and flow development, translation into Portuguese and Spanish, training and retraining (when needed) took about 6 months. The training performed was associated between the match of text generated by the STT module and the triggers associated in the dialog tree flows. The goal was to verify if, from an input presented by the end-user, Pahola was capable of correctly identifying branches and the information associated with the different topics covered in the dialog tree. The dialog tree was structured in 89 core topics (see Figure 1): 49 from the alcohol effects branch, 18 from the quit branch, and 22 from the quiz branch. Approximately 1,500 phrases were used to “train” the NLP. “Small talk” phrases were also added in the script to enhance Pahola's empathy and improve user engagement.

In consultation with PAHO's legal team, a page with terms and conditions was developed, explaining the confidential and anonymous nature of the interactions but requiring access to the video and microphone of the user. Upon acceptance of the terms and conditions, the user could start interacting with Pahola. The page was accessible in any device (mobile, desktop, tablets).

To generate preliminary evidence if the conversational agent could engage users under an anonymous context to disseminate alcohol information, we collected data from the interactions performed by Pahola. Data were extracted from Google Analytics for PAHO's website (https://www.paho.org/en/alcohol/pahola), Google Dialogflow ES and the native software dashboard (which provides overall conversation data, such as number of conversations, average conversation length etc).

In addition, PAHO developed thirteen fact sheets (in English, Spanish and Portuguese) on alcohol-related topics, which were made available through its website, consistent with the information Pahola would be providing. Finally, a communication strategy was developed to increase Pahola's visibility and public awareness in the Region of the Americas. The communication strategy used marketing tactics, including paid ads, to drive people to Pahola's landing page and to raise awareness around health and social problems related to alcohol consumption. The channels used included Twitter, Facebook, Instagram, and Google extended network through DV360 ads. The strategy was complemented by an alcohol awareness and social marketing campaign, focused on alcohol related harms, which was launched two weeks before the launching of Pahola, with the slogan “Live Better, Drink Less”.

On 19 November 2021, Pahola was launched in a webinar with over 1,000 participants (link to the webinar: https://www.paho.org/en/events/launch-meet-pahola-pahos-first-digital-health-specialist-alcohol-use). A media release (https://www.paho.org/en/news/19-11-2021-paho-launches-pahola-digital-health-worker-who-can-help-risky-drinkers-decrease) was prepared and sent to journalists, some of which were given a link to use it in advance of the launch, so they could bring up questions during the event and prepare articles for immediate dissemination. United Nations partners and PAHO's digital media promoted Pahola upon its launch. Various news media in Latin America and the Caribbean featured articles about Pahola.

## Preliminary results

From 19 November 2021 until 16 January 2022, Pahola generated 1,500 mentions on digital channels; 12,000 engagements on PAHO's social media channels with 1.6 million people being directly reached. The Key Performance Indicator (KPI) for the banner campaign was to generate a total of 25,000 clicks. The target click volume was exceeded by nearly a 1000%. The target KPI for the video campaign was exceeded by 291%. The number of impressions from the campaign were 61 million. Impressions are how many times a campaign content is displayed on the social media feeds of the target audience, no matter if it was clicked, engaged with or not. They are considered as an important metric because it tracks the ability to display content in front of the intended audience and the possibility to capture attention to the proposed topic.

Between November 19, 2021 and January 16, 2022, there were 236,000 sessions on Pahola's landing pages in all three languages. Around 179,000 (75.85%) of all sessions were generated from DV360 ads, while 47,240 (20%) came from Instagram, Facebook and Twitter. Over 188,000 users visited Pahola's page. Spanish was the most common language (52%), followed by English (30%) and Portuguese (18%). There were more females (57.8%) than males users (42.2%). Most were between 35 and 44 years old (22%), followed by those from 25 to 34 years (21%).

Around 7,800 people clicked on the button “Let Pahola Help You Today” in the PAHO's landing page. Approximately 1,530 people effectively talked to Pahola on average for five minutes (868 in Spanish, 484 in English, 180 in Portuguese). Over 86% of sessions happened *via* mobile phone, and 10.8% *via* a desktop computer. The top ten countries that engaged with Pahola were the USA, Argentina, Colombia, Canada, Haiti, Dominican Republic, Uruguay, Costa Rica, Brazil and Venezuela.

Regarding users' perceptions about their own drinking, most English users (42%) identified themselves as average drinkers, 35% of Spanish users considered themselves as below average drinkers and 34% as above average, and 49% of Portuguese users as below average drinkers.

**Figure 2:**
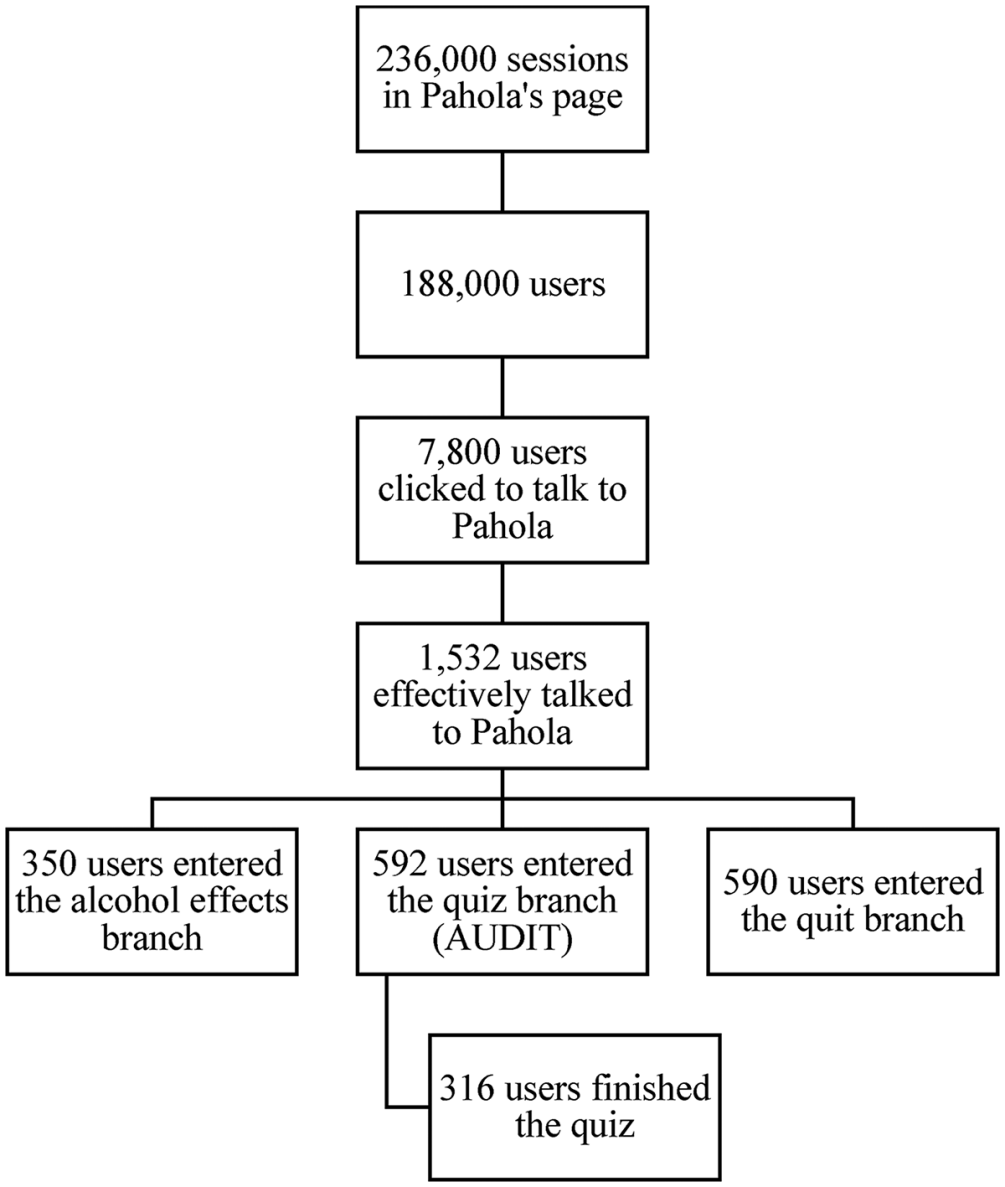
How users accessed Pahola—from November 19, 2021 to January 16, 2022.

Approximately 39% of users in all languages entered the quiz branch (which offered the AUDIT questions) and the same proportion entered the quit branch. Most users interacting in English and Portuguese preferred the quiz branch (41% and 38%, respectively), while most users in Spanish preferred the quit branch (40%). Despite the popularity of the quiz branch across languages, once entered, many users dropped out and not complete the full AUDIT (between 30% and 50% across languages). See Figure 2 for more information.

When looking across languages, the most popular core topics (most accessed) were nutritional value of alcohol, how healthy is to drink, brain and behavior, sex and pregnancy and social drinking. The least visited ones were fetal alcohol syndrome, women's vulnerability to alcohol as compared to men, alcohol and breastfeeding and if alcohol dependence can be cured.

After the quiz, if considered at risk, users could choose between a quit or a cut back plan. Over 50% of all users chose to cut back on their drinking, but there were differences across languages: about 75% of Spanish users made that choice, compared to about 60% of English users and just over 50% of Portuguese users. Of those who entered the quit branch (590 users), more than 90% did not complete it either.

Several questions from users were not answered by Pahola as they were not part of the script (even if the core topic was part of the dialog tree), including specific alcohol effects on the heart, if alcohol is good for you, if there are any benefits from drinking, if alcohol can help fight COVID-19, alcohol and specific types of cancer, causes of alcoholism, how to help an alcoholic, if alcoholism is genetic etc.

## Discussion

Over the last years, PAHO has been providing technical guidance and cooperation to Member States to improve data science in public health using emerging technologies, including artificial intelligence and machine learning, and to foster digital transformation (28). Pahola is the first large-scale and public human-like digital worker focused on alcohol-related topics. It was quickly able to connect to a large worldwide population with state-of-the-art alcohol information, including an assessment of the individual's own alcohol risk and an innovative attempt to improve alcohol health literacy, which could become useful to Member States. In fact, Pahola's capacity to improve alcohol health literacy can already potentially be tested in different countries and settings and it can be done in parallel to its revision by PAHO.

The innovation brought by Pahola is not without challenges. For instance, several unexpected issues led to significant dropouts in the engagement with Pahola. People accessing it from mobile devices, which were the most popular way to do it, reported difficulties finding Pahola on the landing page. Those who clicked to talk to Pahola first had to go over a full page of terms and conditions to provide consent for video and voice access, and that likely discouraged many people to continue. Although the technology behind conversational agents is continuously developed, they currently do not seem to have full human-level language abilities. The impossibility of achieving human level conversational abilities may lead end users to experience misunderstanding and dissatisfaction during the interaction with Pahola. Despite this, the NLP and the NLU technology behind Pahola is the best one currently available.

A positive finding was that most people were interested in assessing their risk and the average time of interactions with Pahola was five minutes, showing a high level of engagement from those who effectively talked to it. On the other hand, it is likely that the quiz was considered too long, justifying their leaving before completing it. Likewise, the few people who got to the quit or cut back plan did not complete it. While we cannot determine the reasons for this drop, a possible reason is that Pahola was not programmed to deliver a complete SBI due to the short time available to develop it before the deployment. The motivational component of the intervention was missing, so Pahola moved from the quiz to proposing a quit or cut back plan without asking users if they wanted to change and were ready for it, which can explain the drop in participation. In addition, in this first version, Pahola's advice may have not been considered fully genuine, as it was not tailored to the user, providing a general guidance to everyone regardless of their drinking levels and situations. Users who were seeking help for a loved one did not obtain a clear answer to what they could do as well.

Therefore, Pahola's continuous development and revision are essential to ensure better usability and engagement. In 2022, Pahola will be available in French and new features are currently being added. The terms and conditions will be optimized to become just a click and interacting with it will be faster and easier for those using mobile devices. Its corpus is being revised in all languages to increment the content on alcohol-related topics, including answers to questions that it could not answer before. New questions considered relevant, such as the role and impact of evidence-based alcohol control policies in enabling healthier choices by all users will also be added to the new version.

In order to address the large drop out with the full AUDIT assessment, we will adopt the short version of the AUDIT (AUDIT-C), which is also an effective and evidence-based screening test for alcohol use, to avoid users' drop off due to the length of the full AUDIT (29). The motivational interviewing component will be added to achieve more tailored and engaging conversations. Pahola will also be able to recall what the user said within the same session, thus facilitating a provision of a summary of what was said, come back to issues raised but not addressed, making it a more personal and engaging experience. New branches on how to help loved ones and for returning users, without violating the anonymity of each interaction, will also be included in the new version.

Artificial intelligence in health care is a fast-evolving field that poses many new challenges (28). The discussion on Pahola's future will consider the constant changes in technology. Creating a framework that could readily determine the most effective and up-to-date version of Pahola could be key to the success of delivering SBI. It should also include considerations of geographical and socioeconomic differences that could impact the level of digital infrastructure, especially in low-income settings, as well as the digital divide and the uneven access to technology by different populations, including health workers. Overall, vulnerable individuals, who can benefit the most from the expanded potential of digital technologies, frequently have the lowest levels of connectivity and digital literacy (30), and may not speak any of the languages currently available for NLP algorithms.

In addition, ethical issues regarding privacy, confidentiality, and concerns of new technology by users and health professionals are constantly being addressed. Ensuring the users' privacy and pursuing their informed consent without limiting Pahola's ability to operate, collect and analyze data is a challenging, but central, task. Risks of flaws, bugs, glitches, and errors in coding and cybersecurity, which are inherent to the technology, must be minimized or eliminated on a regular basis to sustain users' confidence and encourage its use. Our option to use the Google Dialogflow ES for NLP was also based on principles of responsible artificial intelligence practices. Building fair and interpretable algorithms is critical to avoid training bias capable of fostering prejudice or misinformation. There is a well document discussion in the machine learning literature to consider risks associated with discrimination regarding gender, ethnicity, sexual orientation, disability, age, and other socio-demographic factors. The use of responsible practices aims to avoid any type of unfair treatment resulting from using artificial intelligence solutions. The Google Dialogflow ES was developed considering best development practices such as human-centered design, use of multiple metrics to assess training and monitoring, avoidance of creation and reinforcement of bias, and the use of inclusive and interpretable design approaches^1^.

Pahola's future developments will be informed by the needs of national health systems, in joint projects that will gradually inform the next phases of its scalability to provide sustainable benefits to healthcare systems at country level. PAHO is currently discussing a pilot implementation of Pahola in different settings with a few countries. The main goal is to turn Pahola into a global public health good to be used in health systems and in a variety of settings. Its scalability and sustainability depend on providing measurable and long-term impact on the delivery of health programs. At global and regional levels, collaborative efforts towards a less-siloed approach to scaling and integrating digital health may provide the necessary leadership to enable innovative solutions to reach healthcare workers and patients in the Americas. PAHO will continue to improve Pahola and conduct or facilitate research to assess its efficacy and cost-effectiveness.

In conclusion, Pahola is an online tool available to an infinite number of users, 24 h a day, 7 days a week, in three languages, at no cost to end users. It could potentially increase the delivery of SBI, complement health services and ease the burden of health professionals in primary care. It could also improve alcohol health literacy, countering misperceptions of the benefits of alcohol consumption and the lack of clarity on the pervasive harms to drinkers and others. However, its preliminary results pointed to much-needed changes to its corpus and on its accessibility, which are being currently implemented.

The partnership for its development between technology companies and PAHO, as well as a wide-reaching campaign and communication strategy, was fundamental to its acceptability and use. The continuous assessment of Pahola's successes and challenges at different levels, including the inherent technology obstacles that could lead to a digital transformation, is another key element of its potential effectiveness, allowing the ongoing improvement of its capabilities.

## Data Availability

The raw data supporting the conclusions of this article will be made available by the authors, without undue reservation.

## Footnotes

^1^ https://ai.google/responsibilities/responsible-ai-practices/
